# State of the JACMP

**DOI:** 10.1002/acm2.12245

**Published:** 2018-01-15

**Authors:** Michael D Mills

I want to bring the JACMP Community up to date with the evolving status of the JACMP respecting the AAPM and the worldwide clinical physics community. We have just completed a highly successful transition to Wiley and streamlined our publication process. The average number of days to review an article has decreased from 53 to 47 days, and the average number of days from submission to first publication was reduced from 218 to 87 days! Thanks to Wiley for implementing immediate publication of accepted articles. The Impact Factor for the JACMP for 2016 (our last year with Multimed) and released June of 2017 is 1.338; slightly down from the previous year (1.444). One hundred and eighty‐seven articles were published in 2017, down from 248 in 2016. The likely reason for this decline was the implementation of the $500 APC on November 1, 2015. However, the number of submissions to JACMP in 2017 is up by approximately 30% over 2016, so we expect to publish about the same number of articles in 2018 as we did in 2016.

The open access platform of the JACMP continues to support the widespread dissemination of its articles. As of the first of November, and before publication of the 2017 final issue, 30 articles published in 2017 had over 1,000 downloads. The most accessed article (AAPM Medical Physics Practice Guideline 8.a.: Linear accelerator performance tests; Vol 18:4) had 5965 downloads. Clearly, the JACMP is successfully focused on providing clinical articles for the medical physics community without barriers. The JACMP shares some distinctive features with other open access journals. It is much easier to share and use JACMP articles for teaching and operations training. Also, open access journals are experiencing dramatic growth while traditional print‐publication journals are relatively level.

The clinical focus is why the AAPM needs two scholarly journals. The JACMP is focused on clinical, professional, and management articles, mirroring Professional, Education, and Administrative Councils; Medical Physics publishes mostly science articles mirroring the publication focus of the Science Council. However, there is some overlap between the two journals; about one third of the articles in the JACMP could also have been published in Medical Physics. For these articles, the venue of publication is the decision of the authors. For those submitted articles that are not a proper fit for the journal selected by the author, Wiley has now put in place a streamlined method to transfer articles immediately and seamlessly from one journal to the other. Note that the journal Editorial Boards differ in makeup; JACMP Associate Editors and reviewers tend to be clinically focused while Medical Physics editors and reviewers are mostly focused toward scientific contributions.

Clinical medical physicists have publication needs that are different from research medical physicists. An article for clinical physicists should benefit as many patients and as many other clinical physicists as possible, so it makes sense to publish open access. Research medical physics articles may justifiably exist behind a paywall because, other than AAPM members, only selected research physicists have a need for the article and their employers provide the resources to obtain the article. Research physicists want a journal that is more focused on high‐impact science. As such, clinical articles are both a distraction and an unnecessary expense for a purely science journal.

The AAPM's core mission must therefore include publication of both the JACMP and Medical Physics. Each journal fills a unique role to provide publication options for the AAPM members. The Membership Survey of the AAPM, December 15, 2016, (http://www.aapm.org/pubs/MembershipSurvey.asp) supports the JACMP's place as a highly valued initiative of the AAPM. Since it requires both journals to provide the needed publication options and both journals are needed respecting AAPM committee structure operations, both journals should be considered core mission projects of the AAPM.

Finally, with the transition to Wiley, the JACMP has a need to rebuild its reviewer database. I ask all members of the JACMP community to take a few moments and help us by logging in to your profile and complete your areas of expertise. Many thanks for doing this. The instructions are below:

JACMP—Modify Areas of Expertise

Please follow these steps below to login, access the profile screen, and modify the profile screen.

1. Login – https://jacmp.msubmit.net/cgi-bin/main.plex
  - If you have forgotten your Login Name, it is best to send an email to JACMPEditorial@wiley.com
  - If you have forgotten your password, click on “Forgot your password?” just above the Login and Reset buttons on the login page.

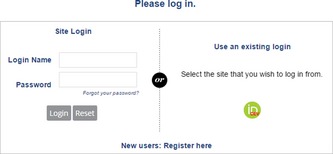


New users will be prompted to fill out affiliation/institution information as well as specifying areas of expertise before the registration process is completed.

- After logging in, the site is separated into several subsections. In the bottom‐most subsection, entitled General Tasks, click on “Modify Profile/Password”.

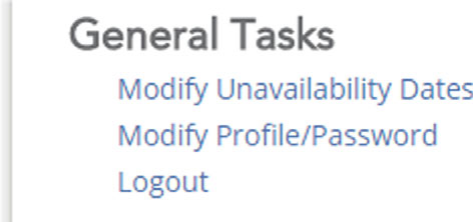

- This is the modify profile page. Here you are able to amend nearly every aspect of your profile and user settings. Scroll down to the bottom of the modify profile page to until you see “Will you consider being a Reviewer for this journal?”
  - Select by clicking on the circle to the left of your answer.

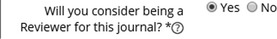

- After the reviewer question, you will see two sections for “Areas of Expertise”. The first section you can search by two methods.
  - Type key words into the “Search Areas of Expertise” text box.
  - Click on “I”, “M”, “T” to manually search the taxonomy.
  - When you find an area you wish to choose simply click on the text. The area you clicked on will be transferred to the box on the right (your selected areas).
- The second section is used for manually entering keywords you cannot find in the first section. Both sections are used by our database when Associate Editors search for potential reviewers.

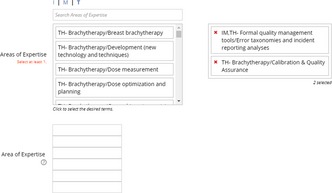


After the fields are modified click on “Modify Profile / Continue” at the bottom of the page to complete the amendments. This is also a good time to update your institutional information and link your existing ORCID profile. Any questions, concerns, or errors while modifying your profile should be sent to JACMPEditorial@wiley.com.

## CONFLICT OF INTEREST

The author declare no conflicts of interest.

